# Supplementary figures and images for: Dupuytren’s Disease Is Mediated by Insufficient TGF-β1 Release and Degradation
Source: Int J Mol Sci. 2023 Oct 11;24(20):15097. doi: 10.3390/ijms242015097 (PMC10606262; doi:10.3390/ijms242015097)

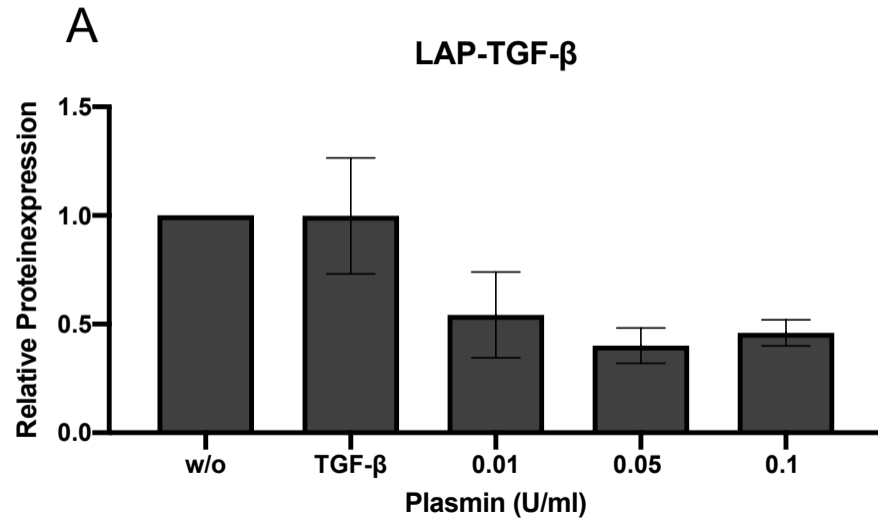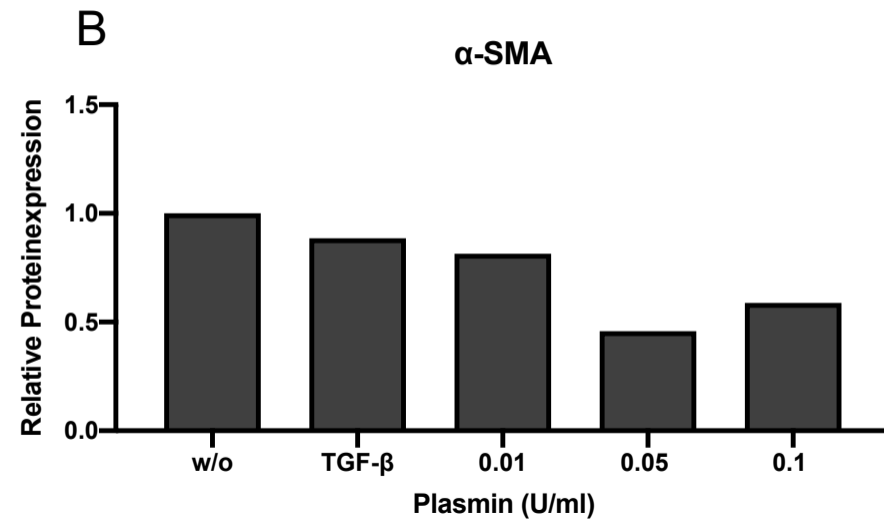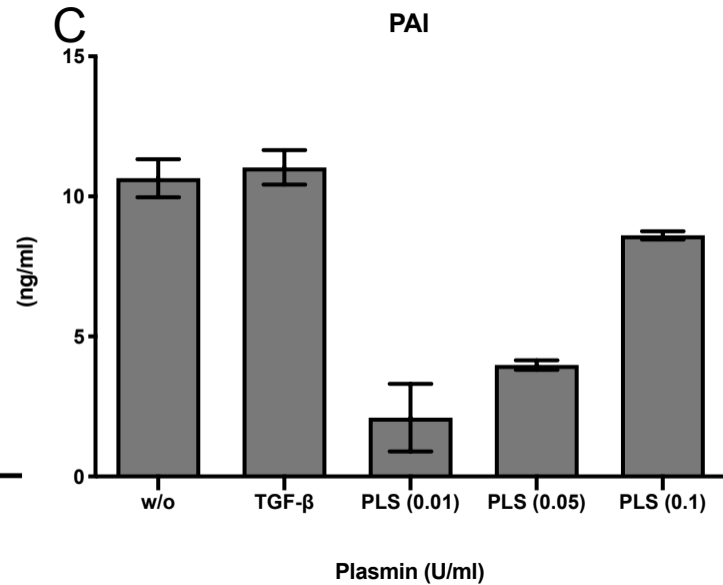

Supplement: Supplementary file 1 [file ijms-24-15097-s001.zip › Supplements S1.pdf]

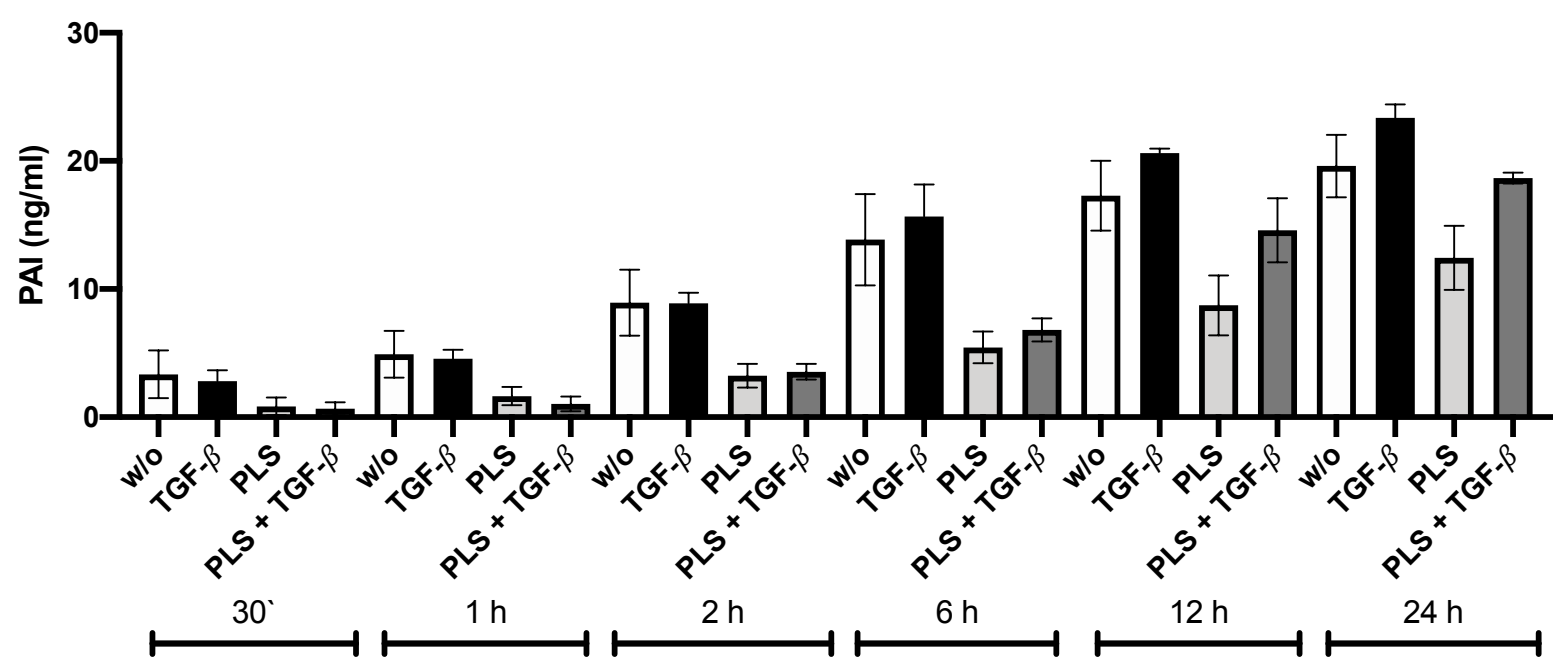

Supplement: Supplementary file 1 [file ijms-24-15097-s001.zip › Supplements S2.pdf]

A

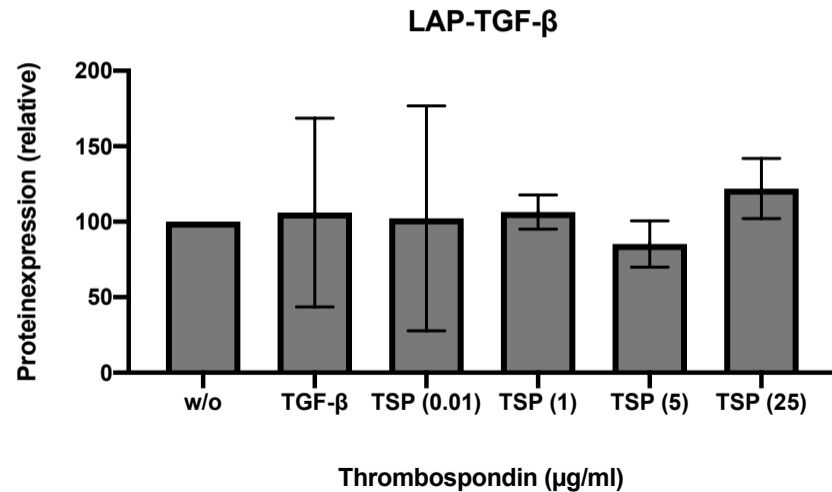

B

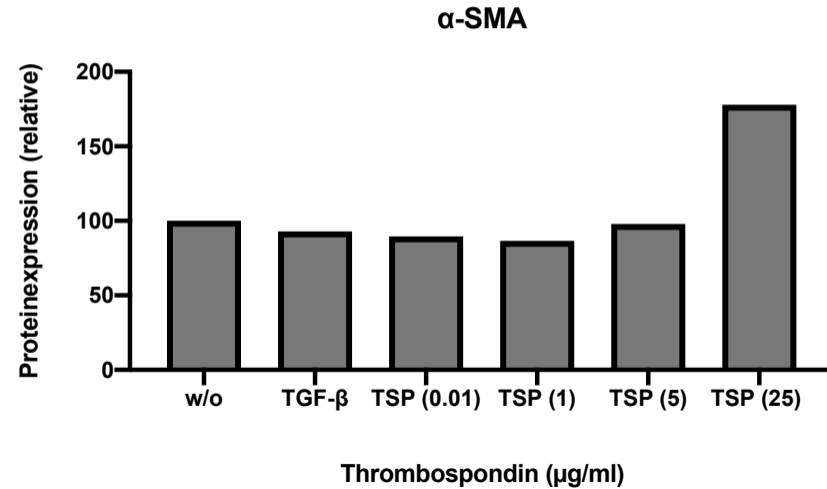

C

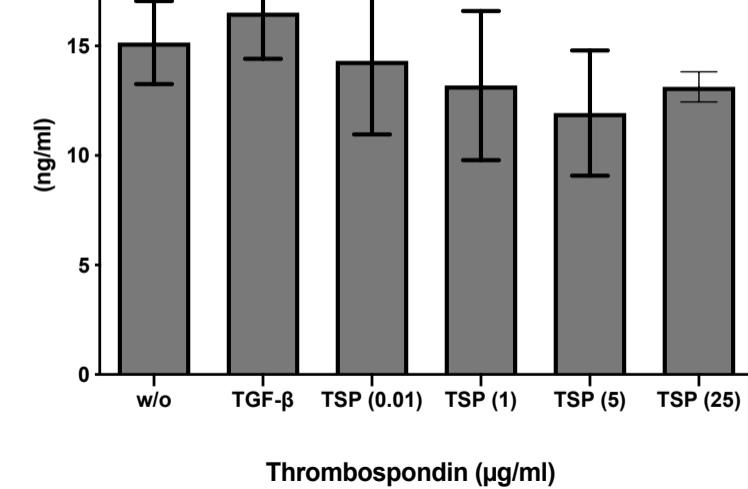

Supplement: Supplementary file 1 [file ijms-24-15097-s001.zip › Supplements S3.pdf]

A

Cell count (relative %)

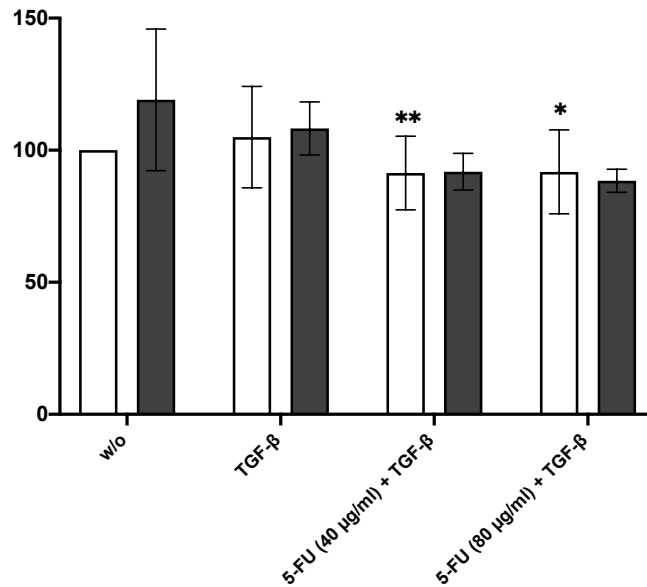

B

Cell count (relative %)

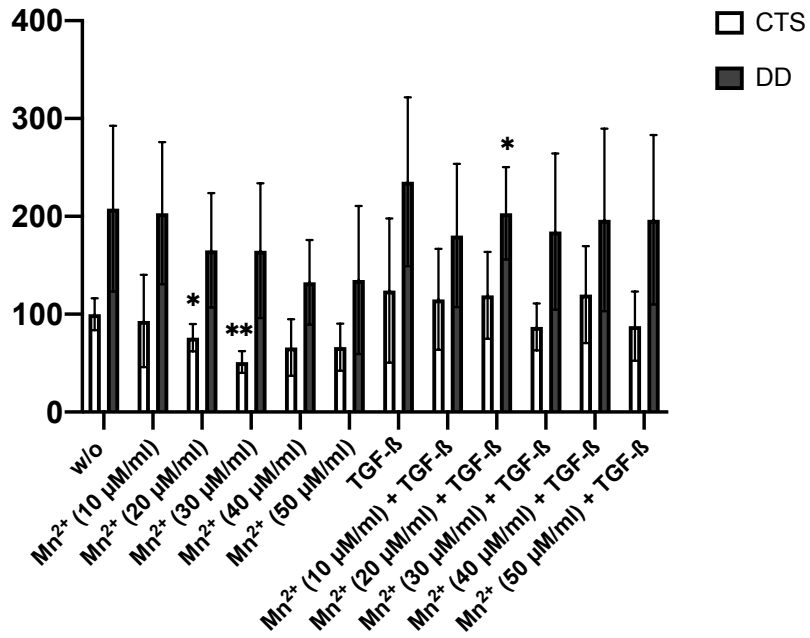

Supplement: Supplementary file 1 [file ijms-24-15097-s001.zip › Supplements S4.pdf]
